# Outpatient antifungal prescribing patterns in the United States, 2018

**DOI:** 10.1017/ash.2021.201

**Published:** 2021-12-22

**Authors:** Kaitlin Benedict, Sharon V. Tsay, Monina G. Bartoces, Snigdha Vallabhaneni, Brendan R. Jackson, Lauri A. Hicks

**Affiliations:** 1 Mycotic Diseases Branch, Division of Foodborne, Waterborne, and Environmental Diseases, Centers for Disease Control and Prevention, Atlanta, Georgia; 2 Division of Healthcare Quality Promotion, Centers for Disease Control and Prevention, Atlanta, Georgia

## Abstract

**Background:**

Widespread inappropriate antibiotic prescribing is a major driver of resistance. Little is known about antifungal prescribing practices in the United States, which is concerning given emerging resistance in fungi, particularly to azole antifungal agents.

**Objective:**

We analyzed outpatient antifungal prescribing data in the United States to inform stewardship efforts.

**Design:**

Descriptive analysis of outpatient antifungal prescriptions dispensed during 2018 in the IQVIA Xponent database.

**Methods:**

Prescriptions were summarized by drug, sex, age, geography, and healthcare provider specialty. Census denominators were used to calculate prescribing rates among demographic groups.

**Results:**

Healthcare providers prescribed 22.4 million antifungal courses in 2018 (68 prescriptions per 1,000 persons). Fluconazole was the most commonly prescribed drug (75%), followed by terbinafine (11%) and nystatin (10%). Prescription rates were higher among females versus males (110 vs 25 per 1,000 population) and adults versus children (82 vs 27 per 1,000 population). Prescription rates were highest in the South (81 per 1,000 population) and lowest in the West (48 per 1,000 population). Nurse practitioners and family practitioners prescribed the most antifungals (43% of all prescriptions), but the highest prescribing rates were among obstetrician-gynecologists (84 per provider).

**Conclusions:**

Prescribing antifungal drugs in the outpatient setting is common, with enough courses dispensed for 1 in every 15 US residents in 2018. Fluconazole use patterns suggest vulvovaginal candidiasis as a common indication. Regional prescribing differences could reflect inappropriate use or variations in disease burden. Further study of higher antifungal use in the South could help target antifungal stewardship practices.

Antibiotic resistance is a serious health problem in the United States. Infections with drug-resistant fungi are a major concern; this includes *Candida* spp, which are becoming increasingly common, and azole-resistant *Aspergillus* infections, which are still relatively rare in the United States but represent a potentially growing threat given the widespread use of azoles.^1^ Reasons for the emergence of antifungal resistance are multifactorial and likely related to antibiotic and antifungal use in humans and environmental fungicides.^2^ Antifungal stewardship is critical because systemic antifungal drug options are limited to only 3 major classes (in addition to terbinafine and griseofulvin, which are not effective against invasive infections), and many of these drugs are expensive and are associated with notable side effects and drug interactions.^3^

Measuring antifungal use is an important initial step in understanding resistance and developing strategies to combat it. Fungal infections vary widely in severity, so antifungal use occurs across many different patient-care settings. Previous analyses have shown that fluconazole accounts for a large proportion of total inpatient antifungal use but that recent declines in its use, combined with increased total antifungal drug costs in community care settings, may indicate a shift toward greater outpatient antifungal use.^3–5^ However, little information is available regarding outpatient antifungal use patterns in the United States. We aimed to describe these patterns to help further inform antifungal stewardship efforts.

## Methods

All antifungal prescriptions dispensed in the United States during 2018 were extracted from the IQVIA Xponent database. IQVIA captures 92% of all retail prescriptions and uses a patented method to project to 100% coverage of all prescription activity based on a comprehensive sample of deidentified prescription transactions, collected from pharmacies that report to IQVIA weekly.^6^ These data represent all retail antifungal prescriptions, across all payers, including community pharmacies and food store pharmacies. The projection method standardizes the data into estimated prescription counts and uses geospatial methods to align the estimated prescriptions for the nonsample pharmacies to prescribers with observed prescribing behaviors for the same product in nearby sample pharmacies. Confidence intervals are not provided because of the high prescription capture rate and fidelity of the projection process.

We grouped antifungal drugs into 5 categories based on the Uniform Classification System^7^ and whether the drug is systemic versus nonsystemic (Table 3). Using Vintage 2018 US Census bridged-race population estimates, we summarized prescription counts and calculated rates per 1,000 population by drug, sex, age group (<20 vs ≥20 years), US Census region, and state where the provider was located.^8^ We also summarized prescriptions by provider specialty based on the American Medical Association self-designated practice specialties, and we used the total number of providers in each specialty from the IQVIA database to calculate specialty-specific rates.

## Results

Healthcare providers prescribed 22.4 million courses of outpatient antifungals dispensed in US pharmacies in 2018 (68 prescriptions per 1,000 persons) (Table 1). Fluconazole was prescribed most frequently (51 prescriptions per 1,000 persons) and accounted for 75% of all antifungal prescriptions, followed by terbinafine (11%) and nystatin (10%). The remaining antifungals together accounted for 3.5% of all prescriptions, including griseofulvin (1.4%), ketoconazole (1%), itraconazole (0.6%), and voriconazole (0.3%).

**Table 1.** Outpatient Antifungal Prescriptions by Age Group, Sex, and Region, United States, 2018

Antifungal Category and Drug	Overall			Age Group^b^		Sex^b^		Region			
	No. of Prescriptions	%^a^	Rate per 1,000 Persons	<20	≥20	Male	Female	Midwest	Northeast	South	West
**Azoles**	17,208,686	76.9	52.6	13.3	65.7	10.8	93.0	52.4	52.4	63.7	35.2
Fluconazole	16,749,502	74.8	51.2	12.2	64.2	9.3	91.8	50.9	51.0	62.3	33.9
Itraconazole	140,998	0.6	0.4	0.2	0.5	0.5	0.4	0.6	0.4	0.4	0.4
Voriconazole	58,668	0.3	0.2	0.08	0.2	0.2	0.2	0.2	0.2	0.2	0.2
Ketoconazole	219,917	1.0	0.7	0.8	0.6	0.8	0.6	0.6	0.6	0.8	0.6
Posaconazole	29,115	0.1	0.1	0.03	0.1	0.1	0.07	0.1	0.1	0.06	0.1
Isavuconazonium sulfate	10,486	0.05	0.03	0.0	0.04	0.04	0.02	0.03	0.05	0.02	0.04
**Echinocandins^c^**	100	0.0	0.0	0.0	0.0	0.0	0.0	0.0	0.0	0.0	0.0
Micafungin	95	0.0	0.0	0.0	0.0	0.0	0.0	0.0	0.0	0.0	0.0
Caspofungin	5	0.0	0.0	0.0	0.0	0.0	0.0	0.0	0.0	0.0	0.0
**Polyenes**	1,788	0.01	0.01	0.0	0.01	0.01	0.0	0.0	0.01	0.01	0.0
Amphotericin B	1,788	0.01	0.01	0.0	0.01	0.01	0.0	0.0	0.01	0.01	0.0
**Other antifungals**	2,873,751	12.8	8.8	4.9	10.1	9.4	8.2	8.6	8.0	9.8	7.9
Flucytosine	700	0.0	0.0	0.0	0.0	0.0	0.0	0.0	0.0	0.0	0.0
Terbinafine	2,559,484	11.4	7.8	1.8	9.8	8.2	7.4	7.8	7.1	8.4	7.5
Griseofulvin	313,567	1.4	1.0	3.1	0.2	1.2	0.8	0.8	1.0	1.4	0.4
**Non-systemic antifungals**	2,298,386	10.3	7.0	8.6	6.5	5.2	8.8	7.7	7.2	7.9	4.9
Miconazole	687	0.0	0.0	0.0	0.0	0.0	0.0	0.0	0.0	0.0	0.0
Nystatin	2,297,596	10.3	7.0	8.6	6.5	5.2	8.8	7.7	7.2	7.9	4.9
Diphenhydramine\lidocaine\nystatin	103	0.0	0.0	0.0	0.0	0.0	0.0	0.0	0.0	0.0	0.0
**Total**	**22,382,711**	**100**	**68.4**	**26.8**	**82.2**	**25.4**	**110.0**	**68.6**	**67.6**	**81.4**	**47.9**

a May not add to 100 due to rounding.

b 0.1% of observations were missing age or sex data.

c No outpatient anidulafungin use was reported.

**Fig. 1.**
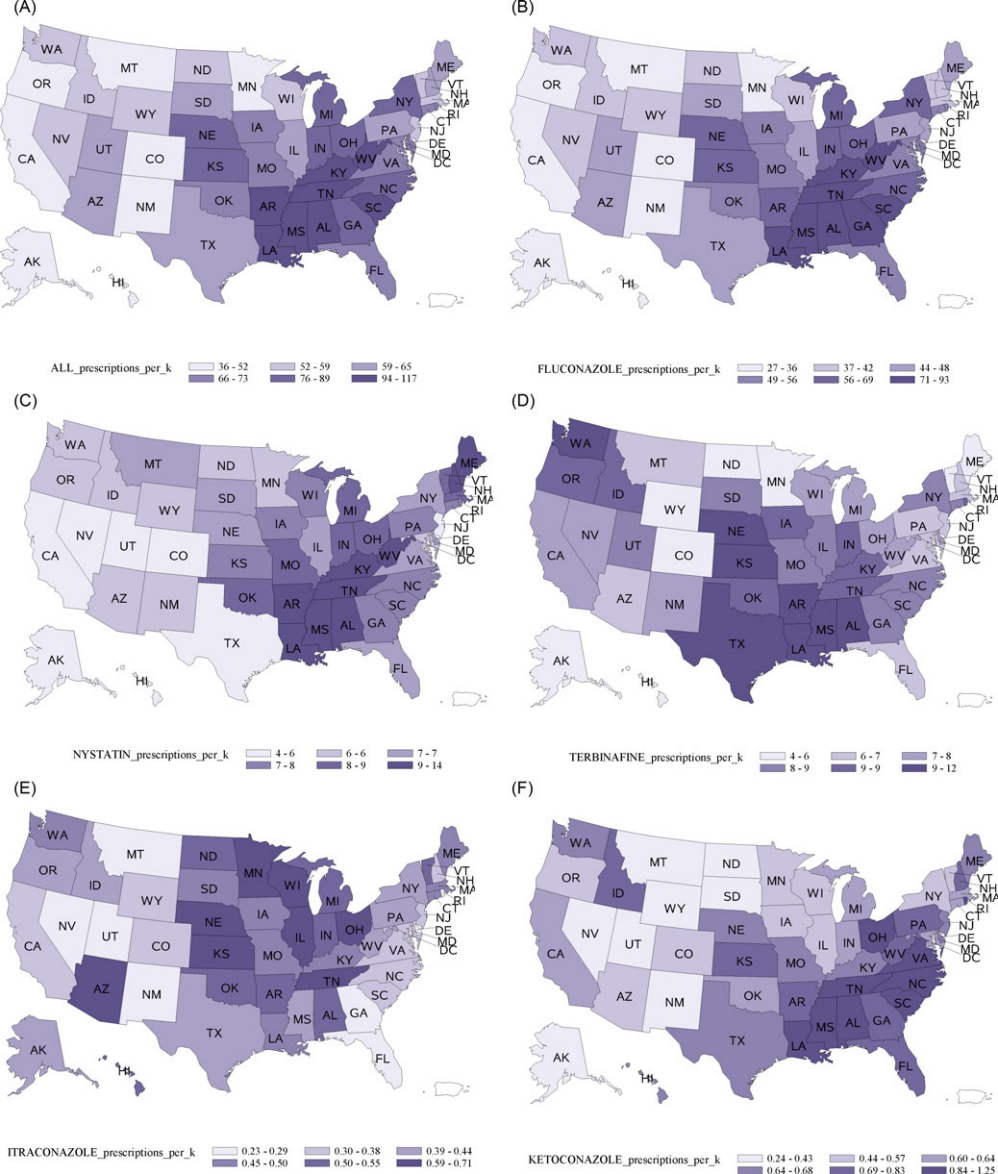
Outpatient prescriptions by state for (A) all antifungals, (B) fluconazole, (C) nystatin, (D) terbinafine, (E) itraconazole, and (F) ketoconazole, United States, 2018.

Females were prescribed antifungals at a rate >4 times greater than males (110 vs 25 per 1,000 population), largely due to differences in fluconazole (92 prescriptions per 1,000 females vs 9.3 per 1,000 males); other antifungals were prescribed similarly by sex. Fluconazole was prescribed more commonly to adults aged >20 years than people <20 years (64 vs 12 per 1,000 population), as was terbinafine (9.8 vs 1.8 per 1,000 population). However, people <20 years had higher rates of nystatin (8.6 vs. 6.5 per 1,000 population) and griseofulvin (3.1 vs. 0.2 per 1,000 population). Overall, antifungal prescription rates were highest in the South (81 per 1,000) and were lowest in the West (48 per 1,000 population). This pattern was consistent across antifungal categories and specifically for fluconazole, terbinafine, and nystatin (ie, the most commonly prescribed drugs); however, itraconazole was more commonly prescribed in the Midwest (Fig. 1).

Nurse practitioners wrote the most antifungal prescriptions (4.9 million), followed by family practice practitioners (4.7 million) and obstetricians/gynecologists (OB/GYNs, 3.1 million). The highest prescribing rates were among OB/GYNs (84 prescriptions per provider) and dermatologists (56 prescriptions per provider) (Table 2). Specifically, fluconazole prescribing rates were highest among OB/GYNs (83 prescriptions per provider), and terbinafine prescribing rates were highest among dermatologists (22 prescriptions per provider). Ketoconazole prescribing rates were highest among dermatologists (2.2 prescriptions per provider) and family practice practitioners (0.6 prescriptions per provider).

**Table 2.** Outpatient Antifungal Prescriptions by Provider Specialty for All Antifungals Combined and for Fluconazole, Terbinafine, and Ketoconazole, 2018

Provider Specialty^a^		All Antifungals		Fluconazole		Terbinafine		Ketoconazole	
	No. of Providers	No. of Prescriptions (%)^b^	Prescription Rate per Provider	No. of Prescriptions (%)^b^	Prescription Rate Per Provider	No. of Prescriptions (%)^b^	Prescription Rate Per Provider	No. of Prescriptions (%)^b^	Prescription rate per provider
OB/Gyn	37,590	3,143,280 (14.0)	83.6	3,109,695 (18.6)	82.7	7,512 (0.3)	0.2	4,724 (2.1)	0.1
Dermatology	11,329	638,056 (2.9)	56.3	318,443 (1.9)	28.1	247,905 (9.7)	21.9	24,468 (11.1)	2.2
Family practice	96,073	4,705,793 (21.0)	49.0	3,383,614 (20.2)	35.2	762,994 (29.8)	7.9	57,086 (26.0)	0.6
Nurse practitioner	109,741	4,921,506 (22)	44.8	4,086,482 (24.4)	37.2	279,273 (10.9)	2.5	19,152 (8.7)	0.2
Physician assistants	63,467	2,238,867 (10)	35.3	1,758,741 (10.5)	27.7	226,531 (8.9)	3.6	16,226 (7.4)	0.3
Internal Medicine/Pediatrics	3,329	111,861 (0.5)	33.6	76,428 (0.5)	23.0	15,458 (0.6)	4.6	678 (0.3)	0.2
Infectious diseases	6,166	179,919 (0.8)	29.2	123,148 (0.7)	20.0	9,855 (0.4)	1.6	508 (0.2)	0.1
Internal medicine	83,841	2,081,644 (9.3)	24.8	1,488,002 (8.9)	17.7	300,487 (11.7)	3.6	20,529 (9.3)	0.2
Otolaryngology	9,536	190,957 (0.9)	20.0	119,196 (0.7)	12.5	829 (0)	0.1	636 (0.3)	0.1
Pediatrics	54,228	862,586 (3.9)	15.9	308,564 (1.8)	5.7	35,394 (1.4)	0.7	3,608 (1.6)	0.1
Urology	10,131	136,284 (0.6)	13.5	131,536 (0.8)	13	783 (0)	0.1	682 (0.3)	0.1
Emergency medicine	32,346	390,027 (1.7)	12.1	324,601 (1.9)	10.0	11,680 (0.5)	0.4	1,941 (0.9)	0.1
Medical subspecialty	74,424	665,330 (3.0)	8.9	460,053 (2.7)	6.2	21,045 (0.8)	0.3	7,518 (3.4)	0.1
Pediatric subspecialty	8,273	30,849 (0.1)	3.7	15,831 (0.1)	1.9	877 (0.0)	0.1	259 (0.1)	0.0
Surgery	69,536	237,433 (1.1)	3.4	189,898 (1.1)	2.7	9,585 (0.4)	0.1	1,579 (0.7)	0.0
Dentistry	122,706	281,462 (1.3)	2.3	190,332 (1.1)	1.6	1,571 (0.1)	0.0	898 (0.4)	0.0
Other	113,783	1,194,244 (5.3)	10.5	388,847 (2.3)	3.4	596,576 (23.3)	5.2	52,772 (24.0)	0.5
**All providers**	**911,814**	**22,382,711 (100.0)**	**24.5**	**16,749,502 (100.0)**	**18.4**	**2,559,484 (100.0)**	**2.8**	**219,917 (100.0)**	**0.2**

a 5,315 providers had missing specialty data.

b May not add to 100% due to rounding.

**Table 3.** Antifungal Drug Type, Category, and Route of Administration

Category	Antifungal Drugs	Route of Administration
**Systemic**		
Allylamines	Terbinafine	Oral
Echinocandins	Anidulafungin	IV
	Caspofungin	
	Micafungin	
Fluorinated pyrimidines	Flucytosine	Oral
Griseofulvin	Griseofulvin	Oral
Imidazoles	Ketoconazole	Oral
Polyenes	Amphotericin B	IV
Triazoles	Fluconazole	IV and oral
	Isavuconazonium	
	sulfate	
	Itraconazole	
	Posaconazole	
	Voriconazole	
**Nonsystemic**		
Imidazoles	Miconazole	Oropharyngeal
Polyenes	Nystatin	Oropharyngeal

## Discussion

This study provides a baseline description of outpatient antifungal use in the United States in 2018, a preliminary step in better understanding where to focus stewardship efforts. Prescriptions for outpatient antifungal drugs were common, with enough courses dispensed for 1 in every 15 residents. Specifically, fluconazole prescribing patterns likely reflect the large public health burden of vulvovaginal candidiasis (VVC) given that OB/GYNs had the highest prescribing rate and females were prescribed fluconazole at a rate 10-fold higher than males. Regional differences in use of azoles and other common antifungal drugs is probably multifactorial and could reflect differences in antifungal prescribing practices, antibiotic prescribing, and fungal disease geography, among other factors. Overall, these results provide a foundation for further studies to understand antifungal prescribing, including what portion may be inappropriate, which will ultimately help inform explore antifungal stewardship strategies and prevent the continued spread of antifungal resistance.

We observed a total outpatient antifungal use rate (68 per 1,000 persons) at an order of magnitude lower than that of antibacterial medications (791 per 1,000 persons in 2018).^9^ Although the volume of antifungal prescribing is substantially lower than that of antibiotics, for which stewardship interventions are more common, large regional variations in antifungal prescribing suggest that antifungal stewardship may also be warranted. Because these data do not allow for evaluation of appropriateness, further study of antifungal prescribing practices is needed to inform interventions. Additionally, the burden of fungal infections relative to other types of infections is not well characterized but is likely far greater than is currently appreciated given the challenges with under diagnosis and lack of comprehensive public health surveillance.^10^ Still, outpatient antifungal use appeared to be common, and this analysis captured only a portion of total US antifungal drug use. For example, many serious invasive fungal infections are both acquired and treated in the inpatient setting (often in intensive care), and approximately half of total antifungal expenditures nationwide attributable to hospitals.^3,5^ Furthermore, over-the-counter (OTC) treatments for superficial infections and VVC are readily available and are used extensively but are not captured in this analysis.^11^ The specific contributions of inpatient, outpatient, and OTC antifungal use to the spread of antifungal resistance are unclear. These concerns are compounded by the reality that there are only 3 primary classes of antifungal drugs used for fungal infections; thus, resistance to one class may render drugs used across settings ineffective.

Although the IQVIA database does not contain information on prescription indications, prescribing of fluconazole by OB/GYNs to adult women appears to reflect treatment for VVC, a common condition estimated to affect up to 75% of women.^12^ This area should be further evaluated for antifungal stewardship efforts because self-diagnosis and empiric diagnosis of VVC are frequently incorrect, leading to inappropriate treatment. Topical OTC agents or single-dose oral fluconazole are recommended for mild-to-moderate infections, whereas severe or recurrent VVC requires treatment with longer courses of fluconazole or other antifungals for azole-resistant infections, which are frequently caused by non-*albicans* species and are a growing public health problem.^13–15^ Higher fluconazole prescriptions per provider among nurse practitioners (n = 37), family practice physicians (n = 35), and physician assistants (n = 28) compared with internal medicine physicians (n = 18), may reflect differences in prescribing for VVC, as well as differences in patient populations and care settings, and warrants further investigation.

Terbinafine, primarily in people aged >20 years, and griseofulvin, primarily in people <20 years, were the most commonly prescribed medications typically used for skin and nail infections, although some nystatin and itraconazole use may reflect such treatment. Ketoconazole was the fifth most commonly prescribed antifungal, which is concerning given that the Food and Drug Administration (FDA) warns that its use is associated with multiple severe adverse effects, including liver failure, and the FDA recommends that it be reserved for serious fungal infections for which no other treatment is available.^16^ More than 200,000 prescriptions were dispensed for ketoconazole in 2018, which suggests that it continues to be widely prescribed for skin and nail infections, despite the FDA warning. Messaging targeting dermatologists and family practitioners may be warranted, given high prescribing rates.

Geographic variability in outpatient antifungal prescriptions was even greater than that reported for antibacterial use, with the South US Census region having an antifungal prescription rate 70% higher than the West region versus 31% for antibacterials.^17^ Differential antifungal prescribing by region could be related to differences in prescribing practices, disease burden, or both, similar to inpatient antifungal use, likely influenced by differences in overall health status and social health determinants.^3,17^ Additionally, antibacterial use, an established risk factor for VVC and other forms of candidiasis, might partly explain the higher fluconazole use rates in the South. Higher rates of ketoconazole prescriptions in the South versus other regions (0.8 vs 0.6 per 1,000 population) support inappropriate prescribing as a driver of regional differences. Higher rates of itraconazole use in the Midwest compared with other areas is consistent with the geographic distribution of histoplasmosis and blastomycosis and could reflect treatment for these diseases.

In addition to the lack of indication information, another limitation of this analysis is the prescription-based (rather than patient-based) nature of the data, which could lead to overestimates of drugs requiring prolonged use. For example, onychomycosis, which can require 3 months of treatment with drugs like terbinafine,^18^ was commonly prescribed in this study.

Future work to characterize total US antifungal use, including inpatient and outpatient prescriptions and indication information, dose and duration, OTC treatment, and environmental fungicides, will help distinguish appropriate prescribing practices from inappropriate ones and help further target antifungal stewardship strategies.
